# Supplementary material for: Capturing an initial intermediate during the P450nor enzymatic reaction using time-resolved XFEL crystallography and caged-substrate
Source: Nat Commun. 2017 Nov 17;8:1585. doi: 10.1038/s41467-017-01702-1 (PMC5691058; doi:10.1038/s41467-017-01702-1)
Supplement: Supplementary file 1 — Supplementary Information [file 41467_2017_1702_MOESM1_ESM.pdf]

Supplementary Information

Supplementary Note 1. **Comparison of the structures of P450nor determined in this study with previously reported structures of heme-NO systems.**

We previously reported a synchrotron structure of the ferric NO-bound P450nor at 100 K that was similar to the damage-free structure by carefully controlling the X-ray dose[1]. However, conventional synchrotron-based crystallography is in principle not a method that can achieve a structure free of radiation damage. In the present study, we characterized the intact ferric Fe-N-O coordination geometry of P450nor with no X-ray damage (i.e., Fe-N-O angle of 158° and Fe-NO bond length of 1.67 Å). The damage-free coordination structure of P450nor agrees with the structure of a thiolate-ligated heme model complex[2]. This is because the structures of model complexes are much less damaged due to the use of an in-house X-ray source and no (or low) content of water responsible for reducing metal centers through forming hydrated electrons. Some of the structures reported for ferric heme proteins, as determined by a synchrotron X-ray source, are different from those of their model compounds, suggesting that they may be more or less affected by X-ray exposure.

In contrast, the ferrous heme proteins and model complexes, both of which are not susceptible to photo-reduction, show a highly bent Fe-N-O geometry (<150°) with an elongated Fe-NO bond length (>1.7 Å), which agrees with the tendency toward structural changes in the damaged form of P450nor. In general, during inevitable X-ray radiation damage, the heme iron is photo-reduced from the ferric to ferrous states. However, in P450nor, the heme absorption spectrum of the damaged form[3] is not the same as that of the ferrous NO form prepared with $Na_2S_2O_4$[4]. The photo-reduction occurred at cryogenic temperature (100 K); therefore, it is plausible that the damaged form could capture the ferrous NO geometry that would be different at room temperature. The structural comparison of the damaged and ferrous NO forms will provide detailed information about radiation damage to the heme-NO structure.

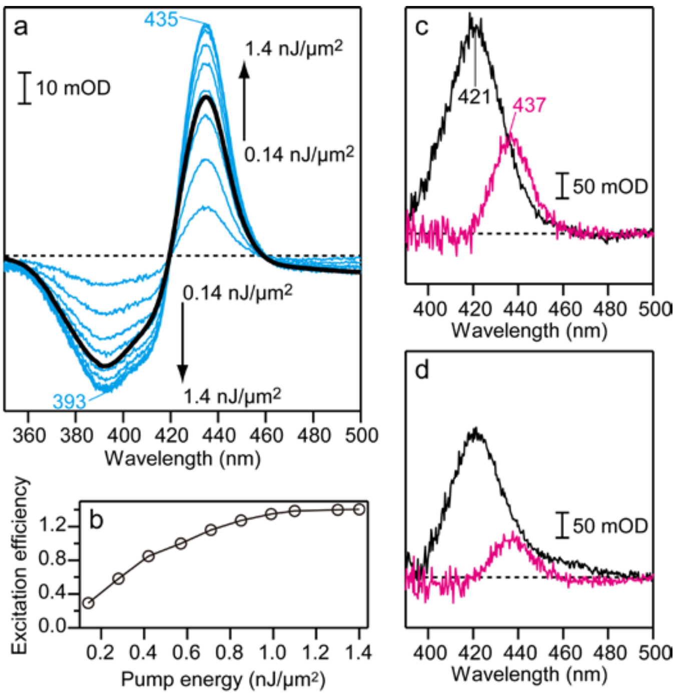

Supplementary Fig. 1. **The quantum yield of NO release from caged-NO with 308 nm pump excitation.** (a) TR visible absorption spectra of P450nor (solution) at a 500 μs time delay with varying pump energies. The difference spectra of "pump-on" minus "pump-off" are shown. The P450nor solution (400 μM at pH 8.5) containing caged-NO (190 μM) was prepared in a cell with an optical path length of 100 μm. The black spectrum is the static difference between the resting state and the NO-bound state prepared with an NO-saturated buffer. (b) Excitation efficiency for caged-NO photolysis. The efficiency was estimated by $|\Delta A_{435} - \Delta A_{393}|$ from the TR difference spectrum, compared with that of the static difference spectrum. The maximum efficiency with sufficient pump energy was 1.4, which corresponds to the quantum yield of NO release from caged-NO. (c, d) TR visible absorption spectra of P450nor (micro-crystals) at a 20 ms time delay. The MC-2 slurry in the absence (c) or presence (d) of the hydroxyethyl cellulose medium was prepared in a cell with an optical path length of 100 μm. Black: absorption spectra of resting P450nor. Magenta: TR absorption difference spectra of "pump-on" minus "pump-off". The intensity ratio of the TR difference peak at 437 nm to the resting enzyme absorption peak at 421 nm was 0.6 ± 0.2 (N = 15) and 0.4 ± 0.2 (N = 15) in the absence and presence of the medium, respectively, which indicates that the medium decreases the generation yield of the NO-bound form by ~33%. This can be explained by the non-transparency of the medium at 308 nm (~0.2 OD with an optical path length of 100 μm). All the measurements were performed at 20 °C. In the solution measurements, the sample was continuously flowed for sample exchange (flow cross section, 150 × 50 μm; flow rate, 2 μL/min), and the pump light illuminated the sample from one direction. The pump and probe repetition rates were 5 and 10 Hz, respectively. In the micro-crystal measurements, the pump light illuminated the sample from two directions (0.51 nJ/μm^2 from each direction), and each TR spectrum was obtained with a single shot. For both measurements, the pump and probe beam sizes were Ø300 and Ø40 μm, respectively.

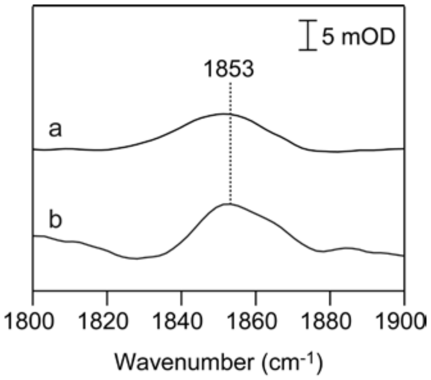

Supplementary Fig. 2. **Static IR spectra of ferric NO-bound P450nor.** The spectra of (a) the MC-1 slurry in the presence of the hydroxyethyl cellulose medium at 293 K and (b) a large single crystal frozen at 100 K are shown. The band observed at 1853 cm^{-1} arises from the NO stretching mode.

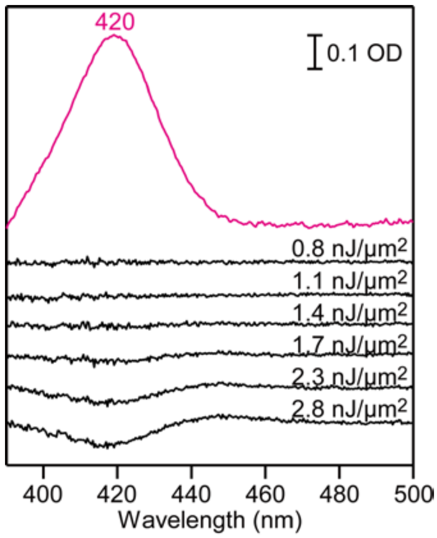

Supplementary Fig. 3. **Visible difference absorption spectra of resting P450nor micro-crystals before and after single-shot UV pulse illumination with various pulse energies.** Absorption spectrum before UV pulse illumination is also shown at the top (magenta). Each spectrum is the average of 5 spectra measured using different micro-crystals. The UV illumination with the pulse energy of >1.4 nJ/µm^2 resulted in a decrease in the heme Soret absorption. The illumination with a higher energy (*e.g.*, ~4 nJ/µm^2) induced crystal cracking. Micro-crystals in the hydroxyethyl cellulose medium were packed in a cell with an optical path length of 100 µm, and the spectral measurements were performed at 20 °C. The nanosecond UV pulse at 308 nm (NT230, EKSPLA) illuminated the sample from two directions, and the indicated pulse energy values are the sum of two.

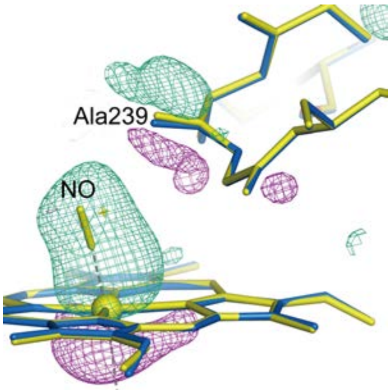

Supplementary Fig. 4. **SFX structures of P450nor in the resting state (blue stick model) and at 20 ms after caged-NO photolysis (yellow stick model).** The F_o("MC-1 Light") − F_o("MC-1 Dark2") difference Fourier maps are shown in turquoise (positive) and magenta (negative) and contoured at 2.8σ.

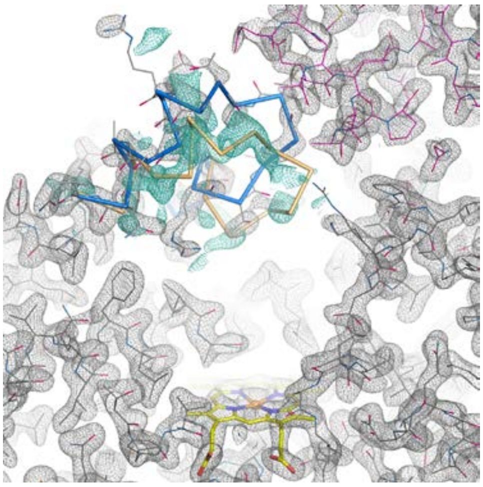

Supplementary Fig. 5. **Structure of the NADH channel entrance in resting P450nor obtained using micro-crystals at 100 K.** The space group of the crystals was $P2_1$. The open (blue Cα trace) and closed (orange Cα trace) conformations are present even at 100 K. The $2F_o - F_c$ maps are shown in gray and contoured at 1.0σ. The $F_o - F_c$ positive maps are shown in turquoise and contoured at 2.3σ. The structure factor F_c was calculated from the open form of the NADH channel. The diffraction data were collected at BL32XU in SPring-8.

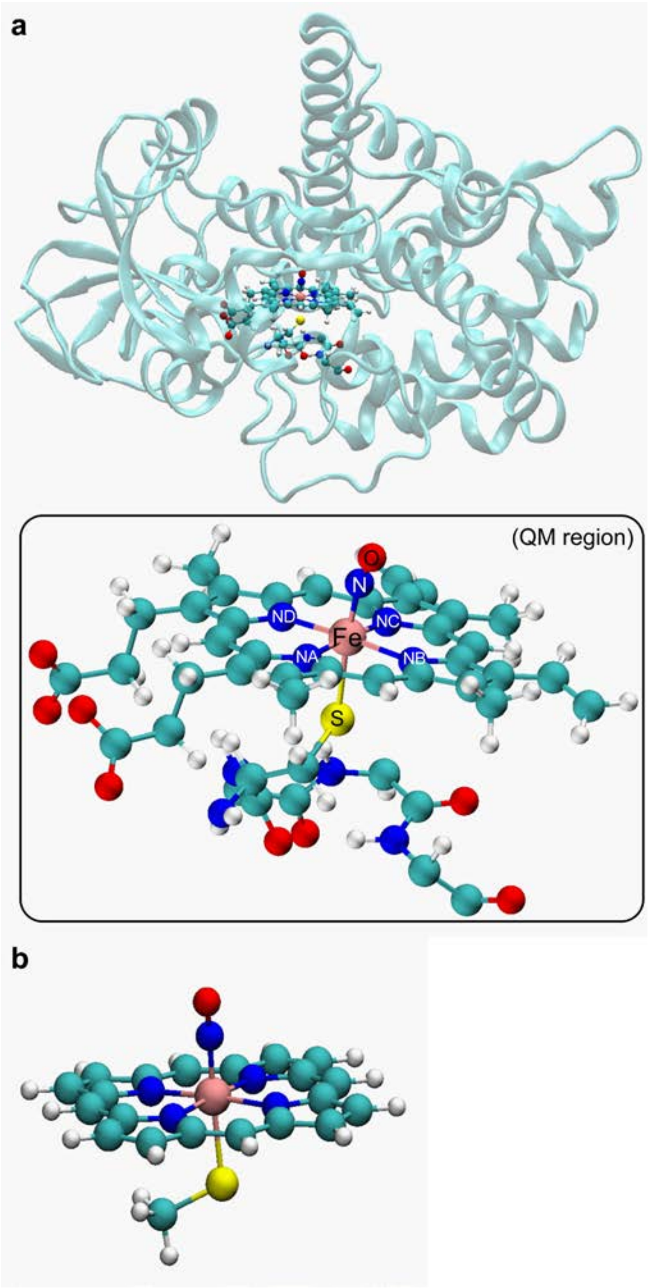

Supplementary Fig. 6. **Calculation models.** (a) QM/MM model of the ferric NO complex of P450nor. The QM region including the heme active site is shown in the inset. (b) Isolated heme QM model.

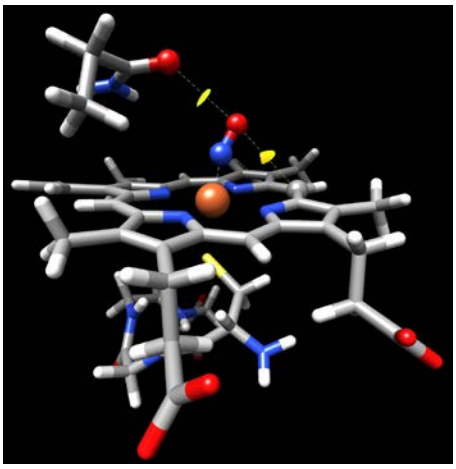

Supplementary Fig. 7. **Non-covalent interactions between NO and its surroundings.** The weak interactions within a radius of 1.7 Å from the NO oxygen atom were visualized by NCIPLOT[5]. To evaluate the interaction between NO and Ala239, the QM region was extended to include Ala239 in the NCI analysis. Yellow regions represent the reduced density gradient isosurfaces (s = 0.5 au) on which the reduced density is less than 0.01 au. Two interaction sites are identified: a meso-carbon atom of the heme and a main-chain C=O oxygen atom of Ala239, which could contribute to the repulsive potentials for Fe-N-O bending in the small and large Fe-N-O angle ranges, respectively.

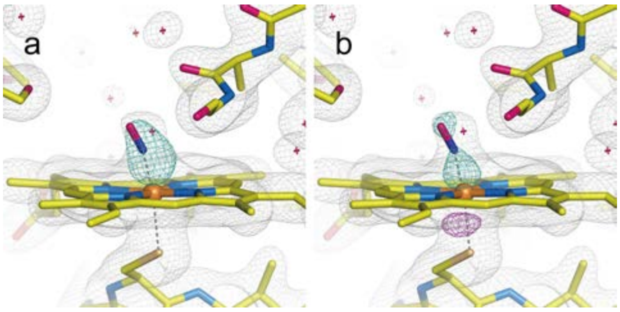

Supplementary Fig. 8. **Difference Fourier maps of the SFX data of MC-1.** (a) The F_o(“Dark1”) – F_o(“Dark2”) and (b) F_o(“Light”) – F_o(“Dark1”) difference Fourier maps are shown in turquoise (positive) and magenta (negative) and contoured at 5σ. The $2F_o - F_c$ maps are shown in gray and contoured at 1.2σ. The structure using the “Light” data of MC-1 is presented.

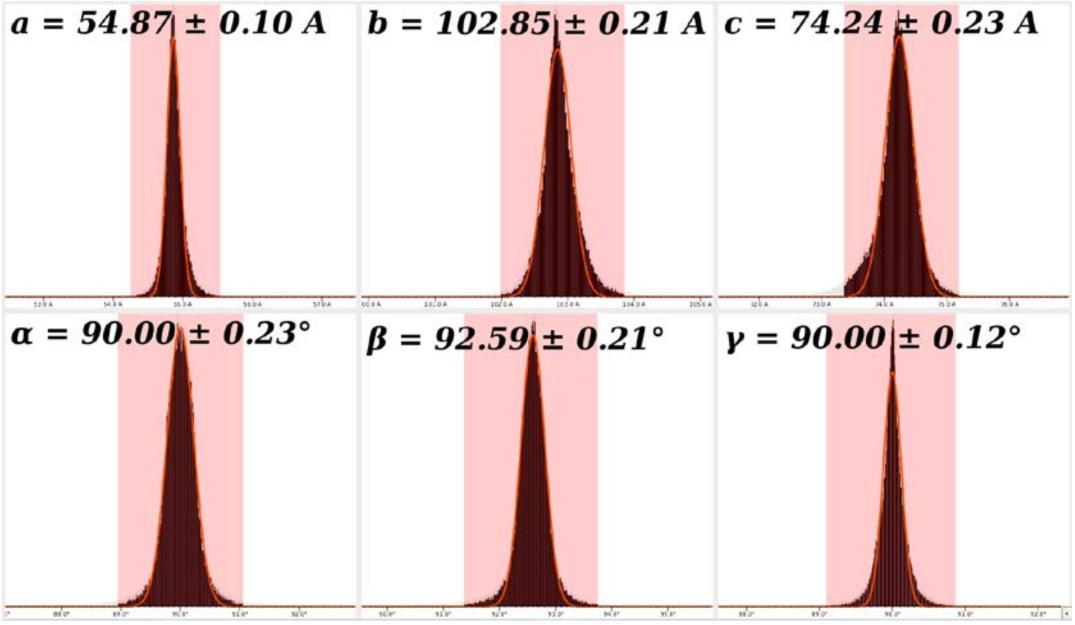

Supplementary Fig. 9. **Distribution of the unit cell parameters of the SFX data.** The "Dark2" data of MC-1 are presented. The data indicate a monoclinic crystal that does not contain a mixture of orthorhombic crystals.

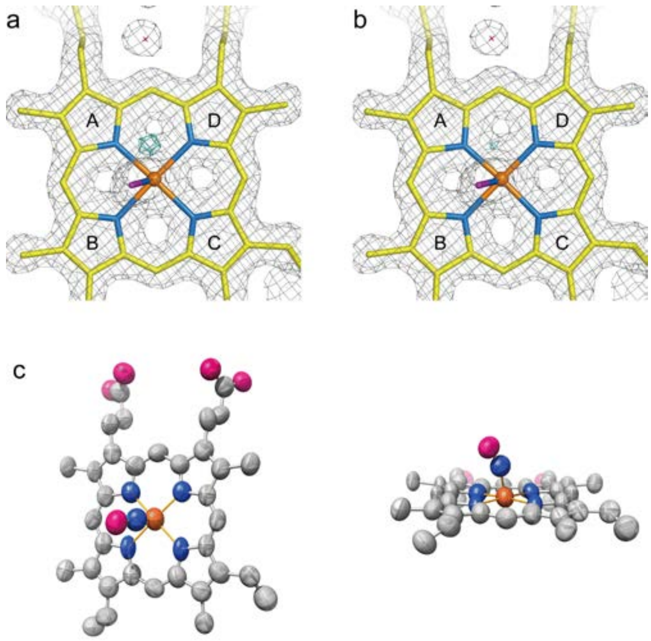

Supplementary Fig. 10. **Structural refinement of the heme.** (a) Isotropic and (b) anisotropic structure refinement of the damage-free form. (c) Displacement ellipsoids at the 20% probability level in the anisotropic refinement. The $2F_o - F_c$ maps are shown in gray and contoured at 1.5σ, whereas the $F_o - F_c$ maps are shown in turquoise (positive) and magenta (negative) and contoured at 3.5σ. The isotropic refinement gave a residual density between porphyrin rings A and D in the $F_o - F_c$ map, whereas the anisotropic refinement showed less residual density and an improved free *R*-factor.

Supplementary Table 1. Geometrical parameters (in Å and °) for NO coordination in the heme-NO species.

	Fe-NO	N-O	Fe-N-O	Ref
(Fe^{3+}-Cys)				
P450nor (SACLA)	1.67	1.15	158	this study
P450nor (SPring-8, low-dose)	1.68	1.19	147	this study
P450nor (SPring-8, high-dose)	2.1	1.42	122	this study
P450nor	1.63	1.16	161	1
P450nor S286V	1.62	1.13	162	1
P450nor S286T	1.65	1.13	165	1
P450cam	1.76			6
Bacillus subtilis NOS	1.69	1.14	161	7
(Fe^{3+}-Tyr)				
Bovine liver catalase	1.87	1.13	159	8
(Fe^{3+}-His)				
Nitrophorin 4	1.66	1.08	156	9
Myoglobin	1.68	1.13	180	10
Leghemoglobin	1.68	1.12	173	11
Arthromyces ramosus peroxidase	1.95	1.21	125	12
(Fe^{3+}-thiolate)				
Fe(OEP)[S-2,6-$(CF_3CONH)_2C_6H_3$]	1.67	1.19	160	2
(Fe^{3+}-neutral ligands)				
Fe(OEP)(1-MeIm)	1.646	1.135	177.3	13
Fe(OEP)(Pz)NO	1.627	1.141	176.9	13
Fe(OEP)(Iz)NO	1.632	1.136	177.6	13
Fe(TPP)(H_2O)	1.65	1.15	174	14
(Fe^{3+}, 5-coordinate)				
Fe(OEP)	1.64	1.11	177	14
(Fe^{2+}-His)				
Sperm whale myoglobin	1.89	1.15	112	15
Leghemoglobin	1.97	1.35	145	16
Cytochrome *c* peroxidase	1.84		135,125	17
Hemoglobin (R-state)	1.8(α), 2.1(β)	1.17(α,β)	138(α), 125(β)	18
(Fe^{2+}, 5-coordinate)				
Cytochrome *c*'	1.84	1.19	142	19
Fe(OEP)	1.722	1.167	144	20
Fe(TPP)	1.739	1.163	144	21

Supplementary Table 2. Experimental and computational geometrical parameters (in Å and °) for the NO coordination in the ferric NO complex of P450nor.

	SF-ROX	QM/MM	Isolated QM	Reduced QM/MM
Fe-NO	1.67	1.67	1.68	1.65
Fe-N-O	158	159	162	164
N-O	1.15	1.15	1.14	1.15
Fe-S	2.33	2.40	2.29	2.40
Fe-NA	1.97	2.02	2.04	2.02
Fe-NB	2.05	2.06	2.04	2.06
Fe-NC	2.06	2.03	2.02	2.03
Fe-ND	1.99	1.99	2.02	1.99

Supplementary References

1. Shimizu, H. *et al.* Proton delivery in NO reduction by fungal nitricoxide reductase. Cryogenic crystallography, spectroscopy, and kinetics of ferric-NO complexes of wild-type and mutant enzymes. *J. Biol. Chem.* **275**, 4816–4826 (2000).
2. Xu, N., Powell, D. R., Cheng, L. & Richter-Addo, G. B. The first structurally characterized nitrosyl heme thiolate model complex. *Chem. Commun.* 2030–2032 (2006).
3. Sakaguchi, M. *et al.* A nearly on-axis spectroscopic system for simultaneously measuring UV–visible absorption and X-ray diffraction in the SPring-8 structural genomics beamline. *J. Synchrotron Rad.* **23**, 334–338 (2016).
4. Shiro, Y. *et al.* Spectroscopic and kinetic studies on reaction of cytochrome P450nor with nitric oxide. Implication for its nitric oxide reduction mechanism. *J. Biol. Chem.* **270**, 1617–1623 (1995).
5. Johnson, E. R. *et al*. Revealing Noncovalent interactions *J. Am. Chem. Soc.* **132**, 6498–6506 (2010).
6. Obayashi, E. *et al.* Unique binding of nitric oxide to ferric nitric oxide reductase from *Fusarium oxysporum* elucidated with infrared, resonance Raman, and X-ray absorption spectroscopies. *J. Am. Chem. Soc.* **119**, 7807–7816 (1997).
7. Pant, K. & Crane, B. R. Nitrosyl-heme structures of *Bacillus subtilis* nitric oxide synthase have implications for understanding substrate oxidation. *Biochemistry* **45**, 2537–2544 (2006).
8. Purwar, N., McGarry, J. M., Kostera, J., Pacheco, A. A. & Schmidt, M. Interaction of nitric oxide with catalase: structural and kinetic analysis. *Biochemistry* **50**, 4491–4503 (2011).
9. Roberts, S. A. *et al.* Ligand-induced heme ruffling and bent no geometry in ultra-high-resolution structures of nitrophorin 4. *Biochemistry* **40**, 11327–11337 (2001).
10. Rich, A. M. *et al.* Determination of the Fe-ligand bond lengths and Fe-N-O bond angles in horse heart ferric and ferrous nitrosylmyoglobin using multiple-scattering XAFS analyses. *J. Am. Chem. Soc.* **120**, 10827–10836 (1998).
11. Rich, A.M. *et al.* Determination of Fe-ligand bond lengths and the Fe-N-O bond angles in soybean ferrous and ferric nitrosylleghemoglobin a using multiple-scattering XAFS analyses. *Biochemistry* **38**, 16491–16499 (1999).
12. Fukuyama, K. & Okada, T. Structures of cyanide, nitric oxide and hydroxylamine complexes of *Arthromyces ramosus* peroxidase at 100 K refined to 1.3 Å resolution: Coordination geometries of the ligands to the haem iron. *Acta Cryst.* **D63**, 472–477 (2007).
13. Ellison, M. K. & Scheidt, W. R. Synthesis, molecular structures, and properties of six-coordinate

[Fe(OEP)(L)(NO)]$^+$ derivatives: Elusive nitrosyl ferric porphyrins. *J. Am. Chem. Soc.* **121**, 5210–5219 (1999).

14. Scheidt, W. R., Lee, Y. J. & Hatano, K. Preparation and structural characterization of nitrosyl complexes of ferric porphyrinates. Molecular structure of aquonitrosyl(meso-tetraphenylporphinato)iron(III) perchlorate and nitrosyl(octaethylporphinato)iron(III) perchlorate. *J. Am. Chem. Soc.* **106** 3191–3198 (1984).
15. Brucker, E. A., Olson, J. S., Ikeda-Saito, M. & Phillips, G. N. Jr. Nitric oxide myoglobin: Crystal structure and analysis of ligand geometry. *Proteins* **30**, 352–356 (1998).
16. Harutyunyan, E. H. *et al.* The binding of carbon monoxide and nitric oxide to leghaemoglobin in comparison with other haemoglobins. *J. Mol. Biol.* **264**, 152–161 (1996).
17. Edwards, S. L. & Poulos, T. L. Ligand binding and structural perturbations in cytochrome *c* peroxidase. A crystallographic study. *J. Biol. Chem.* **265,** 2588–2595 (1990).
18. Yi, J., Soares, A. S. & Richter-Addo, G. B. Crystallographic characterization of the nitric oxide derivative of *R*-state human hemoglobin. *Nitric Oxide* **39**, 46–50 (2014).
19. Manole, A. *et al.* Conformational control of the binding of diatomic gases to cytochrome c'. *J. Biol. Inorg. Chem.* **20**, 675–686 (2015).
20. Scheidt, W. R., Duval , H. F., Neal , T. J. & Ellison, M. K. Intrinsic structural distortions in five-coordinate (nitrosyl)iron(II) porphyrinate derivatives. *J. Am. Chem. Soc.* **122**, 4651–4659 (2000).
21. Silvernail, N. J., Olmstead, M. M., Noll, B. C. & Scheidt, W. R. Tetragonal to triclinic–A phase change for [Fe(TPP)(NO)]. *Inorg. Chem.* 48, 971–977 (2009).
